# Biting midges (Diptera: Ceratopogonidae) as putative vectors of zoonotic *Onchocerca lupi* (Nematoda: Onchocercidae) in northern Arizona and New Mexico, southwestern United States

**DOI:** 10.3389/fvets.2023.1167070

**Published:** 2023-05-15

**Authors:** Chandler C. Roe, Olivia Holiday, Kelly Upshaw-Bia, Gaven Benally, Charles H. D. Williamson, Jennifer Urbanz, Guilherme G. Verocai, Chase L. Ridenour, Roxanne Nottingham, Morgan A. Ford, Derek P. Lake, Theodore A. Kennedy, Crystal M. Hepp, Jason W. Sahl

**Affiliations:** ^1^The Pathogen and Microbiome Institute, Northern Arizona University, Flagstaff, AZ, United States; ^2^School of Informatics, Computing, and Cyber Systems, Northern Arizona University, Flagstaff, AZ, United States; ^3^Navajo Nation Veterinary Management Program, Window Rock, AZ, United States; ^4^Eye Care for Animals, Scottsdale, AZ, United States; ^5^Department of Veterinary Pathobiology, College of Veterinary Medicine and Biomedical Sciences, Texas A&M University, College Station, TX, United States; ^6^Pathogen and Microbiome Division, Translational Genomics Research Institute, Flagstaff, AZ, United States; ^7^U.S. Geological Survey, Southwest Biological Science Center, Flagstaff, AZ, United States

**Keywords:** *Onchocerca lupi*, prevalence, nematodes, Ceratopogonidae, biting midges

## Abstract

*Onchocerca lupi* (Rodonaja, 1967) is an understudied, vector-borne, filarioid nematode that causes ocular onchocercosis in dogs, cats, coyotes, wolves, and is also capable of infecting humans. Onchocercosis in dogs has been reported with increasing incidence worldwide. However, despite the growing number of reports describing canine *O. lupi* cases as well as zoonotic infections globally, the disease prevalence in endemic areas and vector species of this parasite remains largely unknown. Here, our study aimed to identify the occurrence of *O. lupi* infected dogs in northern Arizona, New Mexico, and Utah, United States and identify the vector of this nematode. A total of 532 skin samples from randomly selected companion animals with known geographic locations within the Navajo Reservation were collected and molecularly surveyed by PCR for the presence of *O. lupi* DNA (September 2019–June 2022) using previously published nematode primers (COI) and DNA sequencing. *O. lupi* DNA was detected in 50 (9.4%) sampled animals throughout the reservation. Using positive animal samples to target geographic locations, pointed hematophagous insect trapping was performed to identify potential *O. lupi* vectors. Out of 1,922 insects screened, 38 individual insects and 19 insect pools tested positive for the presence of *O. lupi,* all of which belong to the Diptera family. This increased surveillance of definitive host and biological vector/intermediate host is the first large scale prevalence study of *O. lupi* in companion animals in an endemic area of the United States, and identified an overall prevalence of 9.4% in companion animals as well as multiple likely biological vector and putative vector species in the southwestern United States. Furthermore, the identification of these putative vectors in close proximity to human populations coupled with multiple, local zoonotic cases highlight the One Health importance of *O. lupi*.

## Introduction

*Onchocerca lupi* (Nematoda: Onchocercidae) is an emerging zoonotic filarial nematode that was first described in a gray wolf from Caucasia in the Republic of Georgia in 1967 (1). Within the last decade *O. lupi* has shown to be endemic to regions of the United States (US), Europe, Northern Africa, and the Middle East (2–13). The most important definitive hosts of *O. lupi* are domestic dogs (*Canis lupus familiaris*); however, this parasite has also been reported to infect humans (1, 10, 14–16) and wild carnivores like coyotes (*Canis latrans*) and more rarely, wolves (*Canis lupus*), and cats (*Felis catus*). While the increase in incidence of this parasite has renewed interest world-wide, low occurrence of disease within human populations coupled with *O. lupi* classified as a non-reportable veterinary disease in the United States and the current lack of a commercial diagnostic severely impedes our understanding of disease prevalence and geographic distribution. Due to the implications for both animal and human populations, the purpose of this study was to determine the occurrence of *O. lupi* in companion animal populations on the Navajo Reservation, located in the southwestern United States, as it has overlapping reports of *O. lupi* in humans, dogs, and coyotes (15, 17–19) and determine potential vector species.

While *O. lupi* is considered a vector-borne parasite that is putatively transmitted by dipteran hematophagous insects, as are other congeneric species (20), demonstration of active transmission by a suspected vector species remains absent for this parasite. There is limited knowledge on the life cycle of *O. lupi;* the current dogma is grounded in knowledge from more heavily studied *Onchocerca* species (e.g., *Onchocerca volvulus, Onchocerca ochengi*) which suggest the blackfly *Simulium* spp. (Diptera, Simuliidae) as the putative intermediate host of *O. lupi* (21). Recent research has identified *Simulium tribulatum* as a putative intermediate host for this parasite along the San Gabriel River watershed in southern California (United States), but vector competency has yet to be explored (22). An additional study has identified *O. lupi* DNA sequences acquired from head and bodies of *Simulium griseum*, but has not yet been published in peer-reviewed literature. Subsequent black fly sampling in areas of California failed to detect *O. lupi* DNA but identified DNA of other *Onchocerca* genetic lineages likely associated with wild ungulates (23, 24).

Blackflies, such as *Simulium* spp., are the most widespread of the small biting Diptera (25). The genus *Simulium* can thrive in a wide range of climates from tropical to temperate conditions, but the presence of a running water habitat is a necessary component of their life cycle (25). However, reports of autochthonous *O. lupi* in canines from regions lacking the necessary water habitats required for blackfly breeding suggests the possibility of an *O. lupi* vector outside the Simuliidae family. The principal method for control of vector-borne diseases, such as lymphatic filariasis and malaria, is through vector control (e.g., targeted deployment of insecticides) and requires extensive knowledge of the vector species life cycle (26). There is an urgent need to extend the insect sampling spectrum beyond urban waterways to explain increasing endemic disease in anhydrous regions such as Navajo Reservation (43,452km^2^) in the southwestern United States; this is essential for implementing effective vector control strategies to mitigate *O. lupi* transmission.

The dogma around *O. lupi* considers canid species as definitive hosts and an unknown arthropod species as intermediate host. Sexually mature nematode develop in the sclera of the eye within the definitive host where females release microfilariae into canid skin, predominantly in the head, ears, interscapular, and lumbar regions (21). While the putative simuliid intermediate host takes a blood meal, unsheathed microfilariae are ingested and migrate to the midgut followed by the thoracic muscles and develop from L1 (non-infectious) to L3 (infectious) larvae. Once infectious, the L3 migrates to the mouthparts of the intermediate host, ready to infect a vertebrate host during its next blood meal. It is important to consider that, to date, L3 larvae have not been identified in the head of an intermediate host for *O. lupi*, which would serve as unequivocal biological proof for vector suitability. However, current research from closely related *Onchocerca* species suggests L3 larvae are the only developmental stage present in the head of intermediate hosts (27, 28). Current PCR-based methods for *O. lupi* identification alone cannot differentiate viable from nonviable or immature (L1 and L2) from infective (L3) larvae within wild-caught insects and are therefore insufficient when screening individuals or pools of whole insects as potential intermediate hosts. Therefore, the identification of *O. lupi* DNA within the heads of suspected vectors suggests active transmission as only infectious L3 larva DNA should be present in the head of the arthropod, serving as a molecular confirmation of vector suitability.

Current knowledge regarding the patterns of epidemiology and vector-parasite interactions of *O. lupi* is lacking, which is unfortunate as reported incidence in dogs as well as human cases seem to be rising (2–4, 13, 17). *O. lupi* is considered endemic in canids within the southwestern United States, which includes California, Arizona, New Mexico, and Texas (3, 4, 15, 22). Worldwide, there have been 18 reported human cases of zoonotic *O. lupi*, five of which were in the southwestern United States (9, 10, 13, 17, 19) within the past 12 years (21). These clinical reports coupled with the presence of positive *O. lupi* coyotes in northern Arizona and southern New Mexico (15) indicate a need for increased surveillance of hosts and vectors in regions with overlapping *O. lupi* infections in human, dog, and wildlife populations. Furthermore, identifying where *O. lupi* occurs in the environment is crucial for determining the risk of spread to non-endemic regions as well as determining risk for human populations. In the present study, we conducted surveillance of *O. lupi* in canine populations on the Navajo Reservation, located in the southwestern United States (Arizona, New Mexico, and Utah), and used these data to target geographic locations for collection and molecular screening of hematophagous insects to identify potential vectors of the parasite in this region.

## Materials and methods

### Ethics statement

This study was approved by IACUC of Northern Arizona University (Approved protocol 19-016). Written informed consent was obtained from owners for animal participation. Geographic coordinates for positive dog and insect trap site locations were excluded from this manuscript for privacy purposes.

### Geographic/sampling location(s)

The Navajo Reservation is the largest federally recognized sovereign region retained by the Navajo Nation within the southwestern United States and is located largely on the Colorado Plateau. The Reservation covers 43,452km^2^ spanning the states of Arizona, New Mexico, Colorado, and Utah. The Navajo Reservation varies in altitude (940 to 3,153 m) and encompasses several distinct landscapes with varying climates (29). Regardless of the distinct terrains, water is sparse throughout most of the Reservation (30). Three distinct topographies, each with a different climate and vegetation, makeup the Navajo Reservation: the cold, subhumid climate of the mountains, the intermediate steppe climate, and the warm, arid desert climate. Ponderosa pine-covered high plateaus account for 8% of the region with a reported cold and subhumid climate with lows and highs between −15.6°C and 26.7°C and an annual rainfall of 40.64–68.58 cm (31). With a described climate as cold and dry, the mesas and high plains account for 37% of the region with temperatures ranging between −12.22°C and 31.11°C and annual rainfall between 30.48–40.64 cm and reported vegetation is listed as grasses, sagebrush, and pinyon-juniper (29, 32). Lastly, the warm, arid desert landscape accounts for 55% of Navajo land with temperatures ranging between −11.67°C to 43.33°C and annual rainfall between 17.78–27.96 cm (31). Desert vegetation such as grasses and browse plants are limited (29). A 2010 census reported the total population on the Navajo Reservation as 173,637 people (33) whom are widely dispersed across the reservation in part because of the scarce availability of water (34). This study conducted companion animal and insect sampling within all three climates on the Navajo Reservation.

### Animals and samples

From September 2019 through July 2022, we collected 532 skin tissue samples from companion animals (including both domestic and stray dogs and cats) throughout the Navajo Reservation (Arizona, New Mexico, and Utah) to gain thorough surveillance data. Samples were obtained primarily through spay and neuter clinics as well as from any animal undergoing a surgical procedure with owner consent at three veterinary clinics and a mobile unit. Selection criteria included all animals with a minimum estimated age of 6 months. Upon sample collection, animals in this study did not display clinical disease symptoms typical of canine ocular onchocercosis such as nodule formation. However, animals were selected for this study regardless of their health status. All animal handling and sample collection was in accordance with IACUC regulations. Skin samples were obtained using a disposable 0.2 cm skin punch from the inter ocular frontal area of the head, which was previously identified as one of the predilection areas for presence of *O. lupi* microfilariae on the canine host (35), and immediately fixed and stored in 80% ethanol.

### Molecular screening

Upon receipt in the laboratory, skin samples were stored at 4°C until processing. DNA was extracted from 532 skin samples using the Qiagen Dneasy Blood and Tissue kit with a preliminary overnight incubation at 56°C following manufacturer’s recommendations within the tissue lysis protocol (Qiagen). The conventional PCR (cPCR) screening for *O. lupi* from skin samples was based on the partial sequence of the cytochrome c oxidase gene (COI; COIF: 5′-TGATTGGTGGTTTTGGTAA-3′, COIR: 5′-CATAAGTACGAGTATCAATATC-3′) as described previously (36). The cPCR amplification was carried out with a final 25 μL reaction volume consisting of 1X KAPA 2G Master Mix, 8 μL of genomic DNA, and 100 nM forward and reverse COI primers. The following thermocycler conditions were used: 95°C × 3 min, 35 cycles of (95°C × 15 s, 60°C × 30s, 72°C × 1.5 min), followed by 72°C × 1 min. The presence of PCR product was determined by visualization using gel electrophoresis with a 2.0% TAE agarose gel and sequenced directly with capillary electrophoresis using a BigDye Terminator v3.1 Cycle Sequencing Kit on the 3,130 Genetic Analyzer platform (Applied Biosystems). Fifty-two Forward and reverse sequences were assembled using SeqMan v17 (DNAStar) and were queried with BLASTN (37) against the NCBI Nucleotide database (nt) to confirm their identity as *O. lupi*.

### Insect collection

Insects were collected between May and August 2021 from 15 individual sites spanning Arizona and New Mexico on the Navajo Reservation using BG-Sentinel mosquito traps (Biogents) baited with both CO_2_ and the BG-Lure chemical attractant (Biogents). Funding dictated a single insect trapping season. Twelve of the 15 trap sites were targeted collection points; COI cPCR positive dogs identified from phase 1 of this study informed these locations. Three additional non-informed trap sites were included based on accessibility, permissions, and close proximity to dry creek beds. Due to remote trapping locations, traps generally collected insects continuously for 7 days with nonstop CO_2_ emittance. At the end of 7 days, insects were collected and flash frozen for transport to the laboratory at Northern Arizona University. Traps were moved between the 15 locations throughout the insect trapping season depending on accessibility and permissions. Generally, each trap site collected insects during a single week only, however, when technical difficulties (CO2 emittance issues) arose, traps were left at the same site an additional week. Insects were stored at −20°C until further processing. A Leica S8 AP0 dissecting microscope with an attached Canon EOS Rebel T3 camera was used to process all insects which included morphological examination, counting, photographing, and sorting. The small/min size and great abundance of biting midges at most locations informed insect pooling from the same trap in sets of 50 biting midges. Biting midges were pooled into pools between 2 and 50 insects based on the following criteria: the same species, the same site, the same collection week. Seventy biting midge pools were initially processed as whole insects, however, once positive pools were identified, the remaining 15 pools had DNA extracted from the heads and bodies separately.

### DNA extraction, cPCR, and sequencing

Total DNA was extracted from heads and bodies of Dipteran insects using the Dneasy Blood and Tissue Kit (Qiagen) with the addition of 5 min bead beating at 1,500 Hz followed by an overnight proteinase-K digestion at 56°C following their tissue lysis protocol. Extracted DNA was screened for parasite presence using previously published mitochondrial COI gene primers as described above. All samples were evaluated by gel electrophoresis and samples displaying a band were sequenced directly using Sanger sequencing and sequence data was assembled, queried, and deposited using the same methods listed above.

To molecularly determine Diptera species identification where parasite presence was detected, partial sequence using Diptera universal primers (38) for the COI gene (LCO1490F: 5′-GGTCAACAAATCATAAAGATATTGG-3′ and HC02198R: 5′-TAAACTTCAGGGTGACCAAAAAATCA-3′) and were amplified as with the same parameters of the parasite screening, but with modified cPCR conditions: 95°C × 3 min, 35 cycles of (95°C × 15 s, 55°C × 30 s, 72°C × 1.5 min), followed by 72°C × 1 min. Results were visualized on a 2.0% TAE gel and subsequently sequenced, assembled, and examined using the same methods as listed above. The target amplicon size was 710 bp. Sequences were queried against the NCBI nt database using BLASTN. To further confirm and identify insect species, a database of cytochrome oxidase subunit I sequences downloaded from NCBI was generated with the RESCRIPt QIIME 2 plugin version 2021.11.0 + 3.g8aa880e (39) using the taxonomy ids 7147 (Diptera), 6231 (Nematoda), and 2 (Bacteria). Sequences were removed from the database if they contained 5 or more degenerate bases, 10 or more homopolymers, were <500 or >1,600 nt in length, or did not have a top BLASTX hit to a COI sequence in the Uniprot Reviewed (Swiss-Prot) database (40) downloaded 18 May 2022. Taxonomy was assigned to sequences using this database and the q2-feature-classifier with the classify-sklearn naïve Bayes approach (41) within QIIME 2 version qiime2-2022.2 (42). Diptera COI DNA sequences from positive *O. lupi* insects were deposited to the GenBank sequence database under BioProject PRJNA890670. Sequencing data was deposited in GenBank under the BioProject PRJNA890670.

## Results

Of the 532 animals screened in this study, the initial partial COI cPCR results identified 52 skin samples as positive for the presence filarioid nematode DNA. As the COI cPCR primers used in this study are not species specific for *O. lupi*, these 52 samples of interest were directly sequenced using Sanger sequencing technology to confirm species identity. BLAST results showed 50 of the 52 COI sequences had the closest sequence similarity with 100% identity over a minimum of 411 bases with *Onchocerca lupi* (accession number YP_010142643.1). All 50 *O. lupi* sequences were identical. The overall occurrence of *O. lupi* in companion animals on the Navajo Reservation was 9.4%. Two of the 52 samples had BLAST results with the closest sequence similarity (99.42%–100%) to the canine heartworm, *Dirofilaria immitis* (accession number MT027229.1). Each *O. lupi* positive animal was considered as a local hotspot for insect trapping.

Insect trapping resulted in more than 5,000 insects collected from May 2021 through August 2021. Of these, 1,922 insects (38.44%, 70 pools ranging from 2 to 50 whole insects along with 15 pools and 205 individual insects with heads and bodies processed separately) were extracted and screened for parasite presence. The remaining insects were identified morphologically either as non-biting insects (e.g., fruit flies, etc.) or redundant pools of biting midges from trap sites where *O. lupi* positive insects were already identified. A total of 38 insects (29 bodies and 9 heads) and 19 pools were positive for *O. lupi* from 12 of the 15 trap sites (Table 1). These insects were then molecularly identified using Diptera COI gene primers. Unknown Ceratopogonidae sp. (biting midges) were the most abundant insect trapped and had the highest rates of presence of *O. lupi* DNA; 15 pools across four trap sites were *O. lupi* positive (Table 2). Initially, the size and profusion of biting midge pools imposed pool processing as whole insects; however, once *O. lupi* positive biting midge pools were identified, remaining pools were processed with heads and bodies separated. *O. lupi* DNA was identified in both the head and body in biting midge pools. Three pools of biting midges *Culicoides sonorensis* were identified as *O. lupi-*positive at 3 separate trap sites as well as 1 pool of *Culicoides variipennis* at a single trap site. *O. lupi* DNA was also found in the heads of *Culicoides sonorensis* but was detected at lower concentrations than in the pools of the unknown Ceratopogonidae. A total of 19 stable flies (11 bodies and 8 heads), *Stomoxys calcitrans*, were positive for *O. lupi* DNA at 3 different trap sites. Single insect positives from individual sites included eye gnats of the genus *Hippelates* sp., Tachinidae sp., Anthomyiidae sp., *Coenosia attenuata*, *Lucilia sericata*, Oscinelinae sp., *Delia platura*, *Ravinia errabunda*, and seven single fly, single site undetermined species (Table 2). Two Ceratopogonidae sp. biting midges from a single site were positive for an undescribed species of *Onchocerca.* The highest partial COI identity for this parasite was to *Onchocerca lienalis* (94.4%, accession number KX853325; Supplementary Figure 1). Additionally, DNA of the horse stomach nematode *Habronema muscae* was identified in a single house fly, *Musca domestica*, at a single site.

**Table 1:** Insect trapping site descriptions including water sources and species of positive insects.

Location	Informed	Water source	*Onchocerca lupi* positive species
Site 1*	Yes	Lake, creek, farm line, community reservoir	Ceratopogonidae sp.
Site 2*	No	Dry creek bed	Ceratopogonidae sp., *Culicoides sonorensis,*
Site 3	No	No	-
Site 4*	Yes	Large animal water trough	*Stomoxys calcitrans,* Tachinidae sp.
Site 5*	Yes	Dry creek bed	Unknown sp.
Site 6*	Yes	Large animal water trough	*Stomoxys calcitrans*
Site 7	Yes	Large animal water trough	-
Site 8*	Yes	No	Faniidae sp., Anthomyiidae sp.,
Site 9*	Yes	No	*Stomoxys calcitrans, Lucilla sericata*
Site 10*	Yes	Standing rainwater	*Ravinia errabunda,* Unknown sp.
Site 11*	Yes	No	Hippelates sp.
Site 12*	Yes	No	Ceratopogonidae sp.*, Culicoides variipennis, Delia platura, Coenosia, paradidyma*
Site 13	No	No	-
Site 14*	Yes	Dry creek bed	*Culicoides soroensis*
Site 15*	Yes	Dry creek bed	Ceratopogonidae sp., *Culicoides soroensis,* Oscinellinae sp.

Informed insect trap sites were based on close proximity to an *O. lupi* positive companion animal; non-informed insect trap sites refer to locations with no *O. lupi* positive companion animal near-by and were based solely on permissions to trap on the land. *O. lupi* was found in the heads of *Ceratopogonidae* sp., *Culicoides sonorensis*, *Stomoyxs calcitrans,* and a single *Fanniidae* sp. Locations with * denote *O. lupi* positive insect trap sites.

**Table 2:** Insects positive for the presence of *Onchocerca lupi* along with common names and known ecological traits.

Species	Number of insects	Number of sites	Common name	Known ecological trait
Ceratopogonidae sp.	15*	4	Biting midge	Hematophagous, parasitic as larvae/myiasis, aquatic
*Culicoides sonorensis*	3*	3	Biting midge	Hematophagous, parasitic as larvae/myiasis, aquatic
*Culicoides variipennis*	1*	1	Biting midge	Hematophagous, parasitic as larvae/myiasis, aquatic
*Stomoxys calcitrans*	19	3	Stable fly	Hematophagous, aquatic
Fanniidae sp.	4	1	Little or lesser house fly	Lachryphagy
Hippelates sp.	1	1	Eye gnat	Lachryphagy
Tachinidae sp.	2	2	Parasitic fly	Parasitic as larvae/myiasis
Anthomyiidae sp.	1	1	-	Parasitic as larvae/myiasis
*Coenosia attenuata*	1	1	Hunter fly	Parasitic as larvae/myiasis
*Lucilia sericata*	1	1	Blow fly	Parasitic as larvae/myiasis
Oscinellinae sp.	1	1	Fruit fly	Aquatic
*Delia platura*	1	1	-	-
*Ravinia errabunda*	1	1	Flesh fly	Hematophagous, parasitic as larvae/myiasis, aquatic
Unknown	7	2	-	-

Asterisk* indicates an insect pool containing 2–50 insects.

## Discussion

This study conducted molecular screening of companion animals to identify the occurrence of the filarial nematode *O. lupi* and field surveillance of hematophagous insects to demonstrate possible intermediate hosts *of O. lupi*. This study identified 9.4% occurrence of *O. lupi* in asymptomatic companion animals on the Navajo Reservation in Arizona, New Mexico, and Utah in the southwestern United States from 2019 to 2022. This is the first large-scale study on the prevalence of *O. lupi* in dogs and cats in the US, as most published reports focus on clinical cases, diagnostics, and new geographic records (3, 4, 43–45). Only a few epidemiological studies have been conducted for determining the prevalence of *O. lupi* in canine populations, none of these in North America. Previous studies in Greece, Portugal, and Spain reported positivity rates in dogs ranging from 4.8–8% (43, 44). Interestingly, studies investigating prevalence rates of other *Onchocerca* species demonstrated a correlation between statistically higher prevalence rates and host age (46). Many of our samples were collected from spay and neuter clinics focusing on young animals; because of the heavy sampling of these younger animals, it is possible we are under-estimating the prevalence rate of *O. lupi*, particularly because we lack detailed knowledge of various biological parameters of its life cycle, including pre-patent period and patency. Furthermore, it is known that various *Onchocerca* species have an uneven distribution of microfilariae in the dermis of their hosts (47), which may have generated some false negative results in this study as our *O. lupi* detection relied solely upon microfilariae presence within skin snips from a single anatomic location of companion animal hosts. It is important to consider that the animals that were positive for *O. lupi* appeared to be healthy and did not display symptoms of disease at the time of sample collection. This could be due to their young age combined with *Onchocerca’*s long development period into gravid adults. It is also feasible to consider canines with varying levels of tolerance of *O. lupi* infections. The absence of ocular and skin symptoms in conjunction with cPCR positive tests brings into question the pathogenic role of *O. lupi* within its definitive hosts.

All *O. lupi* positive insects belonged to the Order Diptera; we identified 13 different species (Table 1). Among the 38 positive flies, seven could not be identified to species-level morphologically or molecularly. It is not surprising that this study identified unknown Diptera species on the Navajo Reservation, as to date, no comprehensive seasonal entomological survey has been conducted; however, there have been over 150,000 species described within the Order Diptera (48). It is estimated that 5 million morphologically distinct dipteran species exist but have not yet been described (49). Furthermore, the Sanger sequencing data for four of these unknown flies shows mixed calls across the COI gene indicating mixed samples or contamination. Given that these unknown flies were identified as singletons at single locations suggests it’s unlikely any of these unknown non-biting flies are true vectors of *O. lupi*. Additionally, it is unlikely that the eight *O. lupi* positive singleton non-biting flies (Tachinidae sp., Anthomyiidae sp., *Coenosia attenuata, Lucilia sericata,* Oscinelinae sp.*, Ravinia errabunda, Delia platura,* Hippelates sp.) found at single sites are the vector species responsible for disease transmission on the Navajo Reservation. We hypothesize these flies (Tachinidae sp., *Coenosia attenuate,* Oscinelinae sp.) are either predators of smaller insects and acquired *O. lupi* DNA by feeding on insects that took blood meals from an infected host or may have only fed on serosanguinolent secretions of an infected definitive host (*Lucilia sericata, Ravinia errabunda,* Anthomyiidae sp., *Delia platura*, Hippelates sp.). The four Fanniidae sp. found at a single site have potential as *O. lupi* vectors. Three species of Fanniidae, *Fannia canicularis, Fannia benjamini,* and *Fannia thelaziae,* have been implicated as the vector of the eye worm *Thelazia californiensis* by ingesting L1 larvae while feeding on lacrimal secretions. These larvae mature into L3 infectious larvae within the fly and are transmitted to a new host while feeding on lacrimal secretions (50). However, we hypothesize it is unlikely these serve as competent *O. lupi* vectors as they were present only at a single trap site. Seven cryptic fly species found at two different trap sites were unable to be morphologically or molecularly identified. Morphological identification determined these flies were seven different species and unlikely true vectors of *O. lupi* given their infrequency and non-biting appearance.

In this study, we identified the stable fly, *S. calcitrans* (Diptera: Muscidae) containing *O. lupi* DNA, but its role as a suitable vector remains unclear. Stable flies are known to feed on dogs, particularly dog’s ears causing bleeding, lesions/crusts. This would likely attract other non-blood feeding dipteran for an opportunistic meal of organic matter/nutrients. While stable flies are a disease vector (*Habronema microstoma;* horse stomach worm), they are largely considered deficient biological vectors of disease in comparison to other blood feeding flies (51). To date, pathogen/parasite development or reproduction has not been demonstrated within the stable fly other than the horse stomach worm. However, despite being inefficient biological vectors, as a mechanical vector, stable flies are still culpable for pathogen/parasite transmission. In this study, we identified 19 stable flies, including 11 bodies and 8 heads, as positive for *O. lupi* DNA. Positive stable flies originated from 3 different locations on the Navajo Reservation. We hypothesize the *O. lupi* positive stable flies likely fed on an infected mammal and while they are not biological vectors, it is still plausible they are mechanical vectors. Even so, we expect both human populations and companion animals are at low risk of *O. lupi* transmission from stable flies.

Multiple *Ceratopogonidae* sp. (biting midges) were identified as the most abundant *O. lupi* positive insect in our study (*O. lupi* was present in the heads and bodies) and likely an intermediate host in Northern Arizona and New Mexico. The Ceratopogonidae (Diptera) family, which includes the genus *Culicoides*, commonly called biting midges or “no-see-ums,” are considered hematophagous pests and vector several infectious agents including viruses, protozoans, and filarioid nematodes worldwide (52–54). Biting midges are most notable as vectors of several arbovirus livestock diseases such as bluetongue virus (BTV), African horse sickness virus (AHSV), epizootic hemorrhagic disease virus (EHDV), and Schmallenberg virus (SBV), all of which are of considerable veterinary and economic relevance (55). Importantly, biting midges have been implicated as vectors for several filarial nematodes including *Mansonella ozzardi* (Nematoda: Onchocercidae) with humans as the primary host (56), as well as seven reported species of *Onchocerca* infecting a range of hosts including mammals from the biological families Bovidae, Cervidae, and Equidae (20, 54). The Ceratopogonidae biting midges, which we identify here as potential vectors for *O. lupi*, are widespread throughout the United States (57). When considering the potential of vector-borne disease dissemination into non-endemic regions, it is imperative to examine the potential range expansion of the vector species. In terms of range expansion, there are two aspects of vector dispersal: (1) wing-propelled flight of females to find nearby blood-meals (upwards of 5 km) (58), and (2) wind-borne dispersal from wind streams with potential to spread these vector-borne diseases into non-endemic regions upwards of 200 km away (59). Research has demonstrated recent range expansion of *Culicoides* spp. in the southeastern United States (60), and when coupled with wind expansion potential, highlights the immediate risk for vector establishment, and dissemination of *O. lupi* into non-endemic regions.

We hypothesize that *O. lupi* has adapted to two families of hematophagous Diptera as competent vectors depending on the variable geographic area—Simuliidae and Ceratopogonidae. The Navajo Reservation is bordered by the San Juan River in the north, the Little Colorado River (flow in much of this river is ephemeral) to the south, and the Colorado River to the west (61). Despite these major waterways surrounding the Navajo Reservation, the Reservation itself is vast, with large regions lacking moving water habitats necessary for blackfly development. Transmission of the filarial nematode *M. ozzardi*, first described in 1897, further supports our hypothesis of transmission of *O. lupi* by two families of dipteran vectors (62). *Mansonella ozzardi* is a human parasite transmitted by biting midges (mostly members of the genus *Culicoides*) and blackflies (genus *Simulium*) depending on the endemic location. For example, blackflies of the *Simulium* genus were identified as *M. ozzardi* vectors in Central and South America while *Culicoides* spp. were implicated in transmission in Mexico and several Caribbean islands (62). Additionally, an important factor that further supports our hypothesis is that biting midges are superficial blood feeders, as are black flies, which allows for involvement in transmitting skin-dwelling filarioid nematodes as opposed to solenophagy Diptera that feed directly from blood capillaries of vertebrate hosts (e.g., mosquitoes).

Additionally, a pool consisting of two biting midges from a single site (site 9) showed the presence of parasite DNA in the initial COI cPCR. The COI DNA sequence belongs within the *Onchocerca* genus, however, the species has yet to be described (Supplementary Figure 1). Blast results showed the closest match to this unknown sample was *Onchocerca lienalis* with 94.56% identity. Little is known regarding unknown *Onchocerca* species in wildlife and arthropods (23), however, a more comprehensive examination of unknown *Onchocerca* species within North America is needed to catalog the diversity of both *Onchocerca* and the arthropods vectoring them.

Current *O. lupi* surveillance tools targeting the COI mitochondrial gene are limited to detection and cannot provide insight into population structure, at least within the United States. Two recent studies published the first mitochondrial genome as well the first draft genome for *O. lupi* (18, 63). These studies should facilitate the development of high-resolution surveillance tools for *O. lupi* to understand the genetic diversity and relationships among isolates globally. Further research regarding vector competency of Ceratopogonidae and *Culicoides* species is necessary to confirm their role as intermediate hosts for *O. lupi*. Subsequent knowledge of the vector life cycle is paramount to break the cycle of *O. lupi* transmission on the Navajo Reservation. Mitigation efforts to control the vector species will likely decrease and prevent *O. lupi* disease in companion animals thereby reducing the risk of human infection.

## Conclusion

Our increased surveillance of definitive hosts represents the first large scale study of *O. lupi* in companion animals from an endemic area of the US. We identified a prevalence rate of 9.4% and, from these data points, conducted targeted insect trapping to define putative vectors in the southwestern United States. We found strong evidence that biting midge species may be putative vectors in Northern Arizona and New Mexico. We hypothesize that *O. lupi* has adapted to two families of hematophagous Diptera as competent intermediate hosts depending on the geographic area, Simuliidae and Ceratopogonidae. Further research regarding vector competency of Ceratopogonidae and *Culicoides* species is necessary to confirm their role as intermediate hosts for *O. lupi*.

## Data availability statement

The datasets presented in this study can be found in online repositories. The names of the repository/repositories and accession number(s) can be found in the article/Supplementary material.

## Ethics statement

The animal study was reviewed and approved by IACUC of Northern Arizona University (19-016). Written informed consent was obtained from the owners for the participation of their animals in this study.

## Author contributions

CRo, OH, KU-B, GB, GV, RN, CH, and JS contributed to conception and study design. CRo and CRi created and organized metadata database. CRo and CW performed sequence analyses. MF, JU, TK, DL, OH, KU-B, and GV contributed to sample collection. All authors contributed to manuscript revision, read, and approved the submitted version.

## Funding

This work was supported by the Pacific Southwest Center of Excellence in Vector-Borne Diseases under grant no. (1004601).

## Conflict of interest

The authors declare that the research was conducted in the absence of any commercial or financial relationships that could be construed as a potential conflict of interest.

## Publisher’s note

All claims expressed in this article are solely those of the authors and do not necessarily represent those of their affiliated organizations, or those of the publisher, the editors and the reviewers. Any product that may be evaluated in this article, or claim that may be made by its manufacturer, is not guaranteed or endorsed by the publisher.
