# Supplementary figures and images for: Biting midges (Diptera: Ceratopogonidae) as putative vectors of zoonotic Onchocerca lupi (Nematoda: Onchocercidae) in northern Arizona and New Mexico, southwestern United States
Source: Front Vet Sci. 2023 May 15;10:1167070. doi: 10.3389/fvets.2023.1167070 (PMC10225701; doi:10.3389/fvets.2023.1167070)

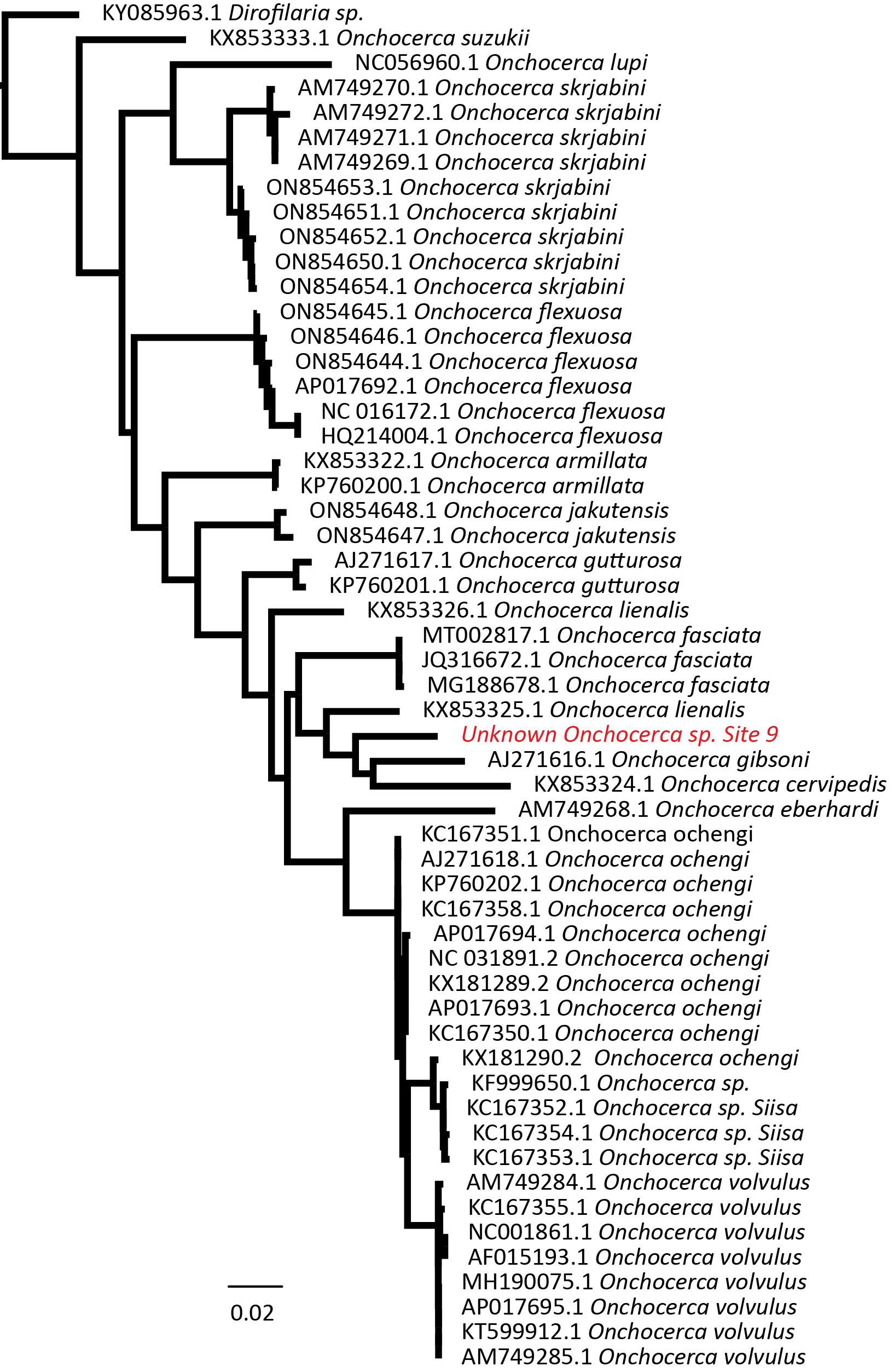

Supplement: Supplementary file 1 [file Image_1.jpg]
